# Death from Hæmorrhage in Gastric Ulcer

**Published:** 1901-03-16

**Authors:** 


### DEATH FROM HÆMORRHAGE IN GASTRIC ULCER.

A little time ago Mr. Mayo Robson delivered an address before the Medico-chirurgical Society of Edinburgh on gastric hæmorrhage and its surgical treatment in which he advanced some rather startling conclusions in regard to the frequency of death from hæmorrhage in cases of gastric ulcer, and founded upon them the opinion that operative procedure should be adopted "after a second bleeding, even during the course of the hæmorrhage, if the patient can stand it, or as soon as his condition will permit the operation to be done." The acceptance of so great an innovation in the treatment of a condition which although admittedly serious is commonly regarded very hopefully by physicians obviously depends upon the statistical proof of the gravity—the hitherto unsuspected gravity—of hæmorrhage in gastric ulcer; and this Dr. Byrom Bramwell[1] questions. He says that if Mr. Mayo Robson's conclusion is accepted and acted upon operations for the arrest of hæmorrhage in cases of gastric ulcer will become of everyday occurrence, "with, I fear, unless the mortality from such operative procedure should in the future become very much less than it is at present, an increase rather than a decrease of the mortality." He goes on to say that statistics, unless the data on which they are compiled are absolutely reliable, are apt to lead to very erroneous conclusions, and he adds that there is, perhaps, no subject in the whole range of medicine on which statistics are more uncertain than the subject of gastric ulcer. Dr. Byrom Bramwell then points out some of the fallacies connected with the statistics on this subject, and endeavours to show that Mr. Mayo Robson has considerably overestimated the total mortality from, and the mortality from hæmorrhage in, gastric ulcer. Finally he says: "Physicians who have had large experience in gastric ulcer will, I fancy, agree with me in thinking that, although the condition of the patient is often most alarming, death from hæmorrhage in cases of gastric ulcer very rarely occurs, and that even in those cases in which repeated hæmorrhages occur it is only in comparatively few cases that the patient dies from the bleeding." This, of course, is the old view, and it was especially to combat this view that Mr. Mayo Robson read his paper. It will then be interesting to see what he will say to the challenge thus thrown down. In fact, it will be more than interesting, it will be most important; for it is to a large extent upon the conviction of the high fatality which attends this disease under ordinary circumstances that Mr. Mayo Robson founds the treatment which he advocates.

[1] Lancet, March 9.

